# Specialized Management of Oral Anticoagulation Therapy Improves Outcome in Patients with Chronic Renal Insufficiency

**DOI:** 10.3390/jcm9030645

**Published:** 2020-02-28

**Authors:** Michael Lauterbach, Eduard Uhrich, Lisa Eggebrecht, Sebastian Göbel, Marina Panova-Noeva, Markus Nagler, Vincent ten Cate, Christoph Bickel, Christine Espinola-Klein, Thomas Münzel, Philipp S. Wild, Jürgen H. Prochaska

**Affiliations:** 1 3rd Medical Clinic-Cardiology, Barmherzige Brüder Hospital, 54292 Trier, Germany; eduarduhrich@web.de; 2 Cardiology I, University Medical Center of the Johannes Gutenberg University Mainz, 55131 Mainz, Germany; sebastian.goebel@unimedizin-mainz.de (S.G.); christine.espinola-klein@unimedizin-mainz.de (C.E.-K.); tmuenzel@uni-mainz.de (T.M.); 3 Preventive Cardiology and Preventive Medicine, Center for Cardiology, University Medical Center of the Johannes Gutenberg University Mainz, 55131 Mainz, Germany; lisa.eggebrecht@uni-mainz.de (L.E.); markus.nagler@unimedizin-mainz.de (M.N.); vincent.tenCate@unimedizin-mainz.de (V.t.C.); philipp.wild@unimedizin-mainz.de (P.S.W.); Juergen.Prochaska@unimedizin-mainz.de (J.H.P.); 4 Center for Thrombosis and Hemostasis, University Medical Center of the Johannes Gutenberg-University Mainz, 55131 Mainz, Germany; Marina.Panova-Noeva@unimedizin-mainz.de; 5 German Center for Cardiovascular Research (DZHK), Partner Site Rhine Main, 55131 Mainz, Germany; 6 Department of Medicine I, Federal Armed Forces Central Hospital Koblenz, 56072 Koblenz, Germany; christophbickel@bundeswehr.org

**Keywords:** oral anticoagulation, vitamin K-antagonists, chronic kidney disease, coagulation service, adverse events

## Abstract

Oral anticoagulation (OAC) is effective at preventing and treating thromboses and thromboembolism in patients with normal renal function. We aimed to research the impact of severe renal failure (RF) on patient outcome and to determine the potential benefit of caring for these patients in a specialized coagulation service (CS). A total of 1516 usual medical care patients and 756 CS-managed patients of the thrombEVAL multicenter (21 centers), prospective, cohort study (NCT01809015) were analyzed in a 3-year follow-up. Patients with RF (serum creatinine >3 mg/dL, no renal replacement therapy) were compared to patients without RF in usual care and a CS. The fluctuations in the international normalized ratios were significantly lower in CS-managed patients, and regardless of treatment in usual care or a CS, the time in therapeutic range was significantly lower in RF patients. Cox regression-adjusted hazard ratios for long-term outcome (1.5, 95% CI: 1.22–1.83, *p* < 0.001), death (1.62, CI: 1.27–2.08, *p* < 0.001), and hospitalization (1.21, CI: 1.02–1.44, *p* = 0.032) were significantly higher in RF patients in usual care. Furthermore, there was a trend of more bleeding events in RF patients. CS-treated patients had significantly lower adjusted hazard ratios for death (0.24, CI: 0.14–0.39, *p* < 0.001), hospitalizations (0.41, CI: 0.34–0.5, *p* < 0.001), clinically relevant bleeding (0.29, CI: 0.18–0.47, *p* < 0.001), and major bleeding (0.33, CI: 0.18–0.59, *p* < 0.001). Thus, patients who required oral anticoagulation therapy benefitted significantly from being managed in a specialized coagulation service, regardless of their renal function.

## 1. Introduction

Oral anticoagulation (OAC) is commonly applied to treat and prevent thromboembolic disease [1,2]. In patients with atrial fibrillation, strokes and their oftentimes debilitating consequences can be effectively prevented with OAC [1]. Vitamin K antagonists (VKAs), such as warfarin or phenprocoumon, were considered the gold standard of effective and efficient stroke prevention for many years until the advent of the new oral anticoagulants (NOACs), and they still represent the first choice for OAC therapy in several countries worldwide [1,3]. Large trials have demonstrated that oral anticoagulation with VKAs is effective and has a limited bleeding risk if managed adequately, even in the elderly [4]. The fixed-dose NOAC evolved as an alternative for traditional VKA therapy for the treatment and prevention of thromboembolism in eligible patients without moderate to severe mitral stenosis or mechanical heart valves [1,5]. Currently, in the setting of impaired renal function, the use of NOACs is preferred over VKAs in eligible patients with a creatinine clearance >25 mL/min but have not been studied in phase III trials in patients with a creatinine clearance below 25 mL/min [6,7,8].

VKAs are still widely used in severe renal failure patients as the risk of stroke or systemic embolism is substantially increased in these patients [9]. However, patients with severe renal failure are often excluded from clinical trials, and thus data for this specific group of patients remain inconclusive and even, in part, contradictory [7]. Interestingly, dialysis patients with atrial fibrillation were paradoxically found to have a higher risk of stroke with the use of warfarin [10,11]. Renal failure accelerates vascular calcification and promotes the progression of cardiovascular disease [12]. Furthermore, renal failure challenges well established cardiovascular treatments, e.g., statin therapy, which, despite its obvious benefits in patients with cardiovascular disease and normal renal function, showed no survival benefit in chronic dialysis patients [13,14].

As data on oral anticoagulation with VKAs in severe renal failure patients are inconsistent and sometimes contradictory, we analyzed data of the thrombEVAL study to determine the impact of severe renal failure on patient outcome. These patients are still widely treated with VKAs as NOACs are not approved for this group of patients. Furthermore, we hypothesized that specific care by a specialized coagulation service would improve the clinical outcome of anticoagulated patients with severe kidney disease.

## 2. Materials and Methods

### 2.1. Study Design and Population

The thrombEVAL study (NCT01809015) is a prospective multi-center investigator-initiated cohort study comprising two prospective cohorts: a cohort of patients having received oral anticoagulation therapy in usual medical care (managed by the physician in charge), and a cohort of anticoagulated patients whose anticoagulation therapy was managed by an eHealth-based, specialized coagulation service, which was provided by an outpatient anticoagulation clinic [15]. The coagulation service was run by trained staff of the Center of Thrombosis and Hemostasis, University Medical Center Mainz, Germany. At 16 service points, CS-enrolled patients had access to standardized visits at fixed consultation hours. The service covered both urban and rural areas. In addition, staff carried out home visits for frail or disabled patients.

Patients for thrombEVAL were recruited from 21 study centers in the federal state of Rhineland-Palatine, Germany, which contains 4.1 million inhabitants. The recruitment period was January 2011 to March 2013. The local ethics committee approved the study (ref. no. 837.407.10.7415/7416), and the independent Interdisciplinary Center for Clinical Studies (Mainz, Germany) monitored the study. The principles of the Declaration of Helsinki and good clinical and epidemiological practice were meticulously followed. The study was registered with clinicaltrials.gov (NCT01809015).

### 2.2. Inclusion and Exclusion Criteria

To be included in the study, patients had to be 18 years old or older, had to give informed consent (or a legal guardian, if appointed), and had to be on oral anticoagulation in the four months preceding the study enrollment. Patients were included regardless of their primary medical problem leading to the reason for hospital admission, and regardless of concomitant medication. Patients in usual care had to be on oral anticoagulation with VKAs for at least 4 months before study enrollment, whereas patients of the coagulation service cohort had to be on oral anticoagulation for 3 months prior to study participation. These requirements were independent of whether patients were self-measuring international normalized ratios (INR) or were physician-managed.

Patients were excluded only if they had contraindications or hypersensitivity to VKAs, or if they participated in other clinical trials. The total dataset comprised 1516 patients in usual medical care and 756 patients managed in a specialized coagulation service, as described elsewhere [16]. For this analysis, patients were separated into two groups based on their renal function. The group with severe renal failure had to have a serum creatinine concentration greater than 3 mg/dL, and the group without severe renal failure had to have a serum creatinine concentration of 3 mg/dL or less (controls). Patients on any intermittent or continuous renal replacement therapy were excluded (see Figure S1, 31 patients in usual care, 2 patients in coagulation service).

### 2.3. Study Procedures

After being included in the study, patients were clinically assessed using standardized case report forms. The recorded data sets comprise medical history, available medical records, laboratory data, current medication, including anticoagulation (a copy of the oral anticoagulation pass comprising information on INR values and the dosing scheme), anticoagulation-associated complications, allergies, sociodemographic data, environmental factors, and the need for nursing care. The study was non-interventional and did not interfere with regular medical treatment.

### 2.4. Outcome Assessment

All study data were subjected to detailed quality control for completeness, plausibility, and validity using pre-specified criteria [15]. During the three-year follow-up period of the study, the incidence of OAC-specific (i.e., bleeding and thromboembolic events) and non-specific (i.e., hospitalization, all-cause mortality) endpoints was recorded via standardized computer-assisted telephone interviews. Source data were gathered to validate information on the study endpoints. Subsequently, all information on adverse events was adjudicated by independent reviewers. 

The primary long-term study outcome was defined as a composite endpoint of stroke, systemic embolism, pulmonary embolism, myocardial infarction, major and clinically relevant non-major bleeding, and death. Secondary outcomes were thromboembolic events, major and minor bleedings, hospitalizations, and all-cause mortality. Major bleeding was defined as a bleeding event with a reduction in a hemoglobin level of at least 20 g/L, a transfusion of at least 2 units of blood, or symptomatic bleeding in a critical area or organ, retroperitoneal bleeding, intra-articular bleeding, or pericardial bleeding [17]. Clinically relevant, non-major bleeding was defined as an event with a mandatory bleeding-associated consultation with a physician in either an ambulatory or clinical setting. Clinically relevant bleeding as an endpoint combined major bleedings and clinically relevant, non-major bleedings. A stroke was defined as the sudden onset of a neurologic deficit with the following characteristics: presumed vascular cause (arterial territory infarction pattern), persistence beyond 24 hours, and absence of other likely causes, such as seizures or tumors. Focal neurologic deficits with symptoms lasting less than 24 hours were considered transient ischemic attacks (TIA) [18]. An intracranial hemorrhage comprised a hemorrhagic stroke, a subdural hemorrhage, and a subarachnoid hemorrhage, while an ischemic stroke with hemorrhagic transformation was not considered as a hemorrhagic stroke.

### 2.5. Statistical Analysis

The discrete variables were presented as absolute and relative frequencies. Normally the distributed continuous variables were summarized with the mean ± standard deviation (SD); the non-normal continuous variables were described using the median with an accompanying interquartile range. Fisher’s exact test was used to test for proportion differences, *t*-tests for two-sided differences in the means of two normally distributed groups, and Mann–Whitney U tests for distributional differences in non-normal variables. The quality of drug treatment was assessed by the calculation of the time in therapeutic range (TTR) according to the linear interpolation method based on INR values [19]. Rate ratios were computed to indicate differences in risk over time between groups. Cox proportional hazards regression models and propensity score matching were used to estimate the difference in time to event between cohorts in multivariable models with an adjustment for potential confounders. A two-sided significance threshold (α) of 5% was used in all statistical analyses. All analyses were carried out in the R environment (version 3.1.1, R Foundation for Statistical Computing, Vienna, Austria).

## 3. Results

### 3.1. Baseline Characteristics of Usual Medical Care Patients with Regard to Renal Failure Status

Of the 1558 study participants in the usual medical care cohort, complete information on the renal function and outcome was available in 1516 subjects after three years of follow-up (Figure S1). The average follow-up time was 2.33 years in usual care and 1.08 years in coagulation service. Only patients with complete follow-up information on renal failure were subjected to further analysis. Of 1516 usual medical care patients, 333 (21.9%) presented with severe renal failure. The median age of the entire cohort was 73.0 years (interquartile range (IQR) = 65–79) with 63.8% male patients.

Subjects with severe renal failure were on average two years older than control individuals without, had a significantly longer experience with oral anticoagulation prior to study enrolment (5.23 vs. 4.36 years, *p* = 0.006), and significantly more home visits (12.7% vs. 5.6%, *p* < 0.001). Table 1 displays the cardiovascular risk factors and comorbidities in patients with and without severe renal failure in usual medical care.

The prevalence of cardiovascular risk factors and comorbidities, such as diabetes mellitus, arterial hypertension, dyslipidemia, coronary artery disease, myocardial infarction, heart failure, atrial fibrillation, chronic lung disease, and liver disease, was significantly higher in patients with severe renal failure (Table 1). The history of thromboembolic disease (i.e., deep venous thrombosis, pulmonary embolism, and stroke) was comparable in both groups, though the CHA_2_DS_2_-Vasc score was significantly higher in severe renal failure patients compared to patients without severe renal failure (controls) (4.72 ± 1.59 vs. 3.95 ± 1.78, *p* < 0.001) (Table 1). However, severe renal failure patients had a higher rate of reported bleedings before being included in the study and, in line with this, a significantly higher HAS-BLED score.

Concomitant medication was comparable in both groups, with beta blockers being the most frequently used medication, followed by lipid-modifying drugs and angiotensin-converting enzyme (ACE)-inhibitors. Only the proton pump inhibitor use was significantly greater in patients with severe renal failure (39.3% vs. 29.8%, *p* < 0.001). There was a non-significant trend towards a higher prevalence of the use of beta blockers (66.1% vs. 60.7%) and calcium antagonists (24.0% vs. 20.4%) in patients with severe renal failure compared with those without. The antiplatelet agent use did not differ between patients with and without renal failure (23.4% vs. 20.4%, *p* = 0.22). None of the patients had dual antiplatelet therapy in addition to oral anticoagulation therapy.

Among the indications for oral anticoagulation, atrial fibrillation was the leading indication in both groups ahead of venous thromboembolism (Table 2). 

Consistent with their significantly higher burden of atrial fibrillation (Table 1), significantly more patients with severe renal failure were on oral anticoagulation for AF compared to controls (Table 2).

### 3.2. Quality of Oral Anticoagulation Therapy

The time in therapeutic range (TTR) was significantly lower in patients with severe renal failure compared with controls (66.59% (47.55/83.42) vs. 71.37% (53.26/86.60), *p* = 0.039). The rate of self-measurement, though, was similar in both groups (15.0% vs 14.8%, *p* = 0.93). Patients with self-measurement among severe renal failure patients (*n* = 50) showed a significantly better TTR (84.31% (69.38/99.38) vs. 63.36% (41.74/80.97), *p* < 0.001) compared to physician-managed patients of the same group. The fluctuations in TTR (instability criterion) were similar in both groups (Stable INR, severe renal failure patients: 67.9%, controls: 74.2%, *p* = 0.062).

### 3.3. Clinical Outcome by Renal Failure Status

**Figure 1:**
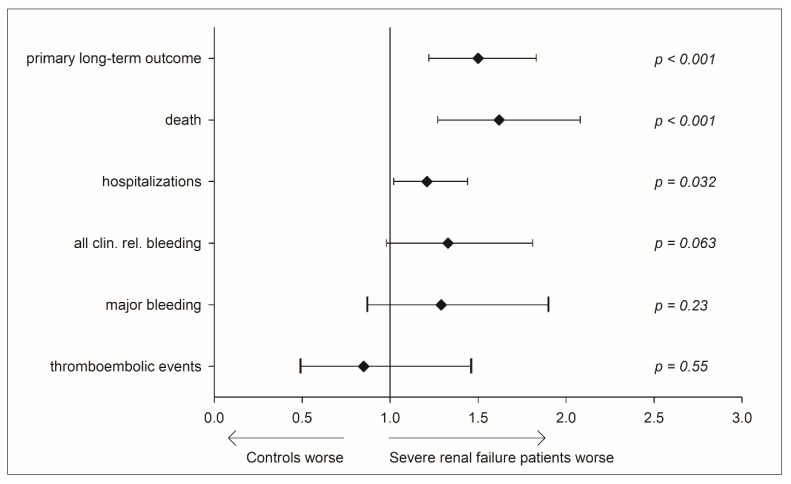
Adjusted safety endpoints and outcome measures comparing severe renal failure patients and controls in usual medical care, Cox regression. Forest plot showing the rate ratios and 95% confidence intervals of endpoints; primary long-term outcome: composite endpoint of clinically-relevant non-major and major bleeding, myocardial infarction, systemic and pulmonary embolism, stroke, and death. Adjusted for age, sex, diabetes, dyslipidemia, hypertension, obesity, family history of myocardial infarction, smoking, atrial fibrillation, congestive heart failure, coronary artery disease, previous myocardial infarction, deep vein thrombosis, pulmonary embolism, and liver disease.

Patients with severe renal failure had a significantly worse clinical outcome at the end of the 3-year follow-up period. The unadjusted rate of clinically relevant bleeding in severe renal failure patients was 1.52-fold (CI: 1.19–1.94, *p* < 0.001), in major bleeding it was 1.55-fold (CI: 1.11–2.13, *p* = 0.0097), and in hospitalization it was 1.36-fold (CI: 1.23–1.50, *p* < 0.001) higher compared to controls. The rate of unadjusted all-cause death was increased 2.18-fold (CI: 1.72–2.74, *p* < 0.001) in severe renal failure patients compared with controls. In a Cox regression analysis, adverse events were investigated under consideration of potential confounding by the concomitant clinical profile by the adjusting for age, sex, diabetes, dyslipidemia, hypertension, obesity, family history of myocardial infarction, smoking, atrial fibrillation, congestive heart failure, coronary artery disease, previous myocardial infarction, deep vein thrombosis, pulmonary embolism, and liver disease (Figure 1).

Compared to controls, individuals with severe renal failure experienced a 50% elevated risk for the primary long-term outcome (HR 1.5, 95% CI: 1.22–1.83, *p* < 0.001) and a 62% increased risk of death (HR 1.62, CI: 1.27–2.08, *p* < 0.001). Hospitalizations were increased by 21% (HR 1.21, CI: 1.02–1.44, *p* = 0.032). In addition, a non-significant trend for all clinically relevant bleedings (HR 1.33, CI: 0.98–1.81, *p* = 0.23) and a higher major bleeding risk (HR 1.29, CI: 0.87–1.90, *p* = 0.063) of individuals with severe renal failure compared with subjects without was observed.

The Cox regression adjustment for antiplatelet use had no significant effect on major bleeding (HR 1.4 (0.84/2.32), *p* = 0.19) or all clinically relevant bleedings (HR 1.43 (0.97/2.12), *p* = 0.074). The propensity score matching did not affect these results (not shown). We then sought to address whether severe renal failure patients would nevertheless benefit from being cared for in a specialized coagulation service.

### 3.4. Relevance of Health Care Setting—Usual Medical Care vs. the Specialized Coagulation Service

In the cohort of 760 patients managed in a specialized coagulation service, complete information on renal failure and outcome was available for 756 patients. Of these, 118 had severe renal failure (Figure S1). The characteristics of the coagulation service cohort by renal failure status are provided in the table in the supplement (Table S1).

Subjects in usual medical care and coagulation-service-managed patients did not differ in age; however, the rate of male patients was significantly higher in usual medical care (63.8% vs. 51.8%, *p* < 0.0001). The time in therapeutic range was significantly higher in coagulation-service-managed patients with 75.41% (61.73/85.82) over 70.61% (50.98/85.95), *p* < 0.001. In line with this, the INR stability was significantly higher in patients cared for in a specialized coagulation service (87.1% vs. 72.8%, *p* < 0.001), whereas no difference existed within each cohort.

Subjects in usual medical care and a coagulation service differed in the family history of myocardial infarction (40.5% vs. 30.3%, *p* < 0.001) and dyslipidemia (54.8% vs. 41.8%, *p* < 0.001). Interestingly, the rate of self-measurement was lower in coagulation service-managed patients, with 9.5% compared to 14.8% in usual medical care, but comparable within each cohort regardless of the renal failure status. Concomitant medication was comparable in both cohorts, with beta blockers being the most frequently used medication, followed by lipid-modifying drugs and angiotensin-converting enzyme (ACE)-inhibitors. In the usual medical care cohort, the use of beta-blockers was significantly higher compared to the coagulation service-managed cohort (61.9% vs. 56.3%, *p* = 0.011). Further digitalis and non-steroidal anti-inflammatory drugs were more frequently used in the usual medical care cohort.

When only comparing patients with severe renal failure in usual care and a coagulation service, patients in the coagulation service were significantly older, and the percentage of female patients was higher. Furthermore, patients with severe renal failure in the coagulation service had diabetes, dyslipidemia, a history of myocardial infarction, congestive heart failure, peripheral artery disease, prior bleeding, and liver disease less frequently when compared with severe renal failure patients in usual care. The prevalence of a history of pulmonary embolism was significantly higher in severe renal failure patients in the coagulation service compared with severe renal failure patients in usual care. Overall, severe renal failure patients in the coagulation service cohort were sicker than their matching severe renal failure cohort receiving usual care.

Atrial fibrillation was the leading indication for oral anticoagulation in both cohorts and did not differ significantly between the severe renal failure patients of both cohorts (usual medical care: 72.1% vs. the specialized coagulation service: 68.6%, *p* = 0.48). Notably, severe renal failure patients had a significantly higher burden of atrial fibrillation compared to their respective controls (Table 1 and Table S1). Comparing only patients with renal failure in both cohorts, the TTR was 71.73% (54.75/83.61) vs. 66.59% (47.55/83.42) in favor of coagulation-service-managed patients (*p* = 0.016). However, in a Cox regression analysis, the TTR was not an independent risk factor for any of the outcome measures.

**Figure 2:**
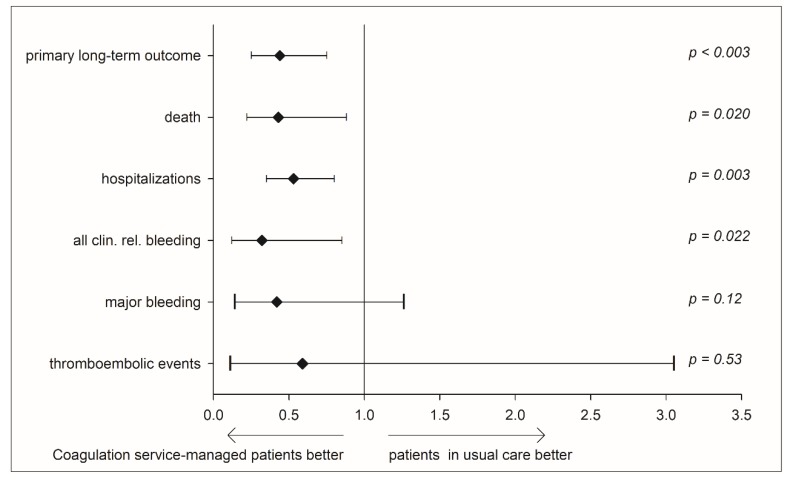
The adjusted safety endpoints and outcome measures comparing severe renal failure patients in a coagulation service with those receiving usual medical care, Cox regression. Forest plot showing the rate ratios and 95% confidence intervals of endpoints; primary long-term outcome: composite endpoint of clinically relevant non-major and major bleeding, myocardial infarction, systemic and pulmonary embolism, stroke, and death. Adjusted for age, sex, and cardiovascular risk factors.

For individuals with severe renal failure, a significantly lower rate of adverse events was observed in the coagulation service cohort as compared with the usual care cohort (Figure 2, unadjusted: Figure S2): the primary long-term outcome (HR 0.44, CI: 0.25–0.75, *p* = 0.003) and incidence of all-cause deaths were 56% lower (hazard ratio (HR) 0.44, CI: 0.25–0.75, *p* = 0.003). Furthermore, hospitalizations (HR 0.53, CI: 0.35–0.8, *p* = 0.003) and clinically relevant bleedings (HR 0.32, CI: 0.12–0.85, *p* = 0.020) were significantly lower. Due to the limited sample size and event frequency, no statistically relevant differences were detected for major bleeding (HR 0.42, CI: 0.14–1.26, *p* = 0.12) and thromboembolic events (HR: 0.59, CI: 0.11–3.05, *p* = 0.53).

### 3.5. Usual Medical Care vs. the Specialized Coagulation Service—Adjusted Comparison

In a Cox regression analysis, adverse events in both cohorts (usual medical care and the coagulation service) were investigated under a consideration of potential confounding by the concomitant clinical profile by age, sex, diabetes, dyslipidemia, hypertension, obesity, a family history of myocardial infarction, smoking, atrial fibrillation, congestive heart failure, coronary artery disease, previous myocardial infarction, deep vein thrombosis, pulmonary embolism, liver disease, and renal failure. In all outcome measures, coagulation-service-managed patients fared significantly better than patients in usual medical care, regardless of their renal failure status at the end of the 3-year follow-up period (Figure S3) [17]. In detail, CS-treated patients had significantly lower adjusted hazard ratios for death (0.24, CI: 0.14–0.39, *p* < 0.001), hospitalizations (0.41, CI: 0.34–0.5, *p* < 0.001), clinically relevant bleeding (0.29, CI: 0.18–0.47, *p* < 0.001), and major bleeding (0.33, CI: 0.18–0.59, *p* < 0.001). Thromboembolic events were lower, but the reduction just missed statistical significance (0.52, CI: 0.27–1.00, *p* = 0.050). The propensity score matching did not alter the results.

**Figure 3:**
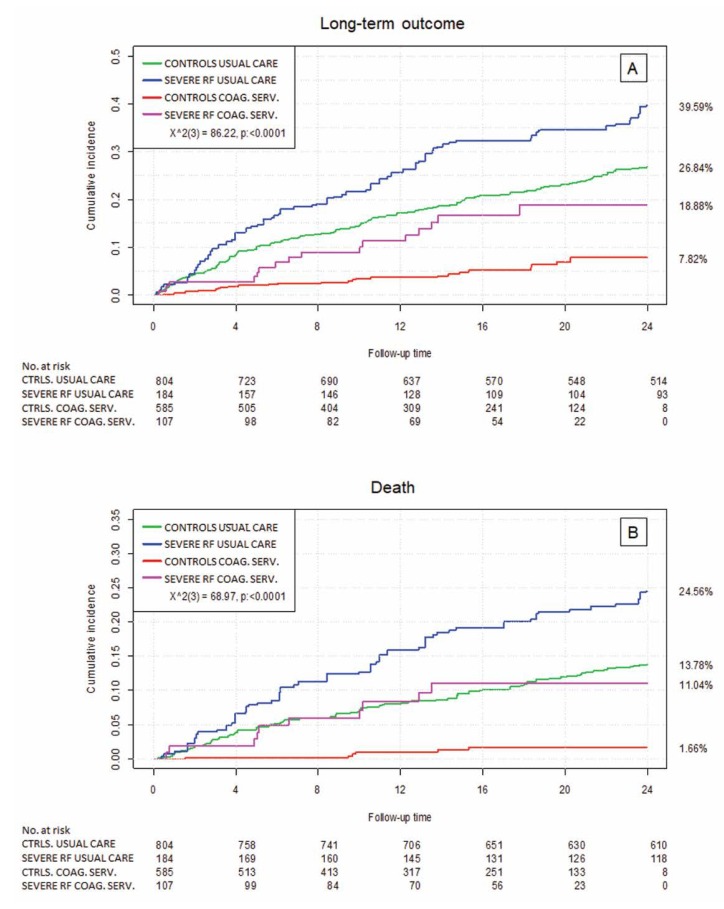

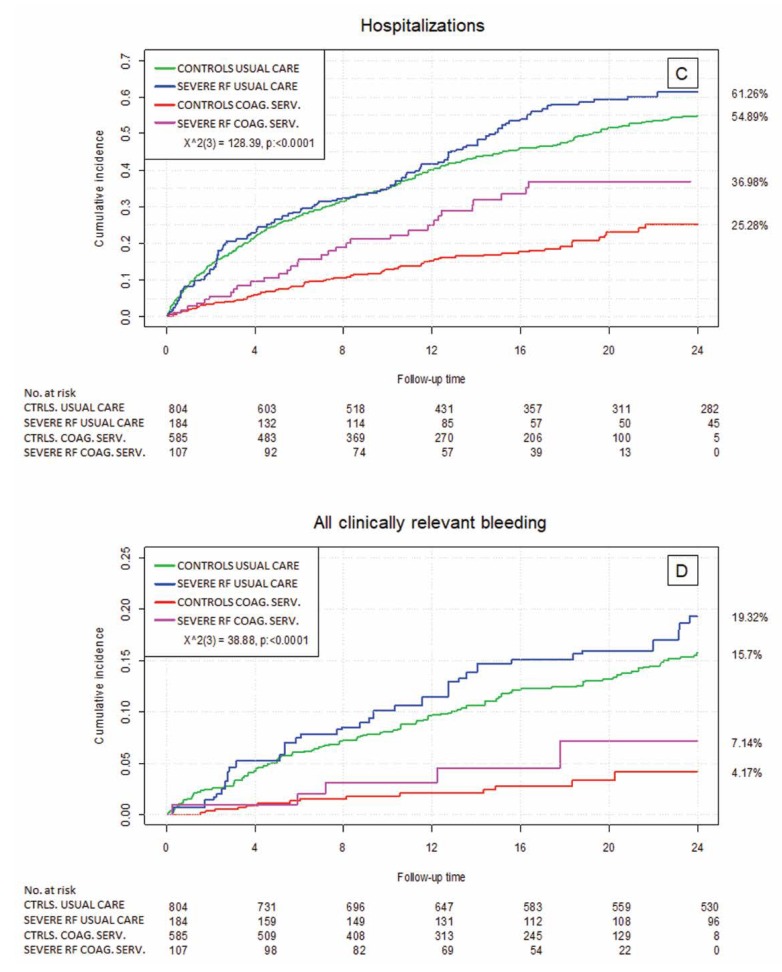

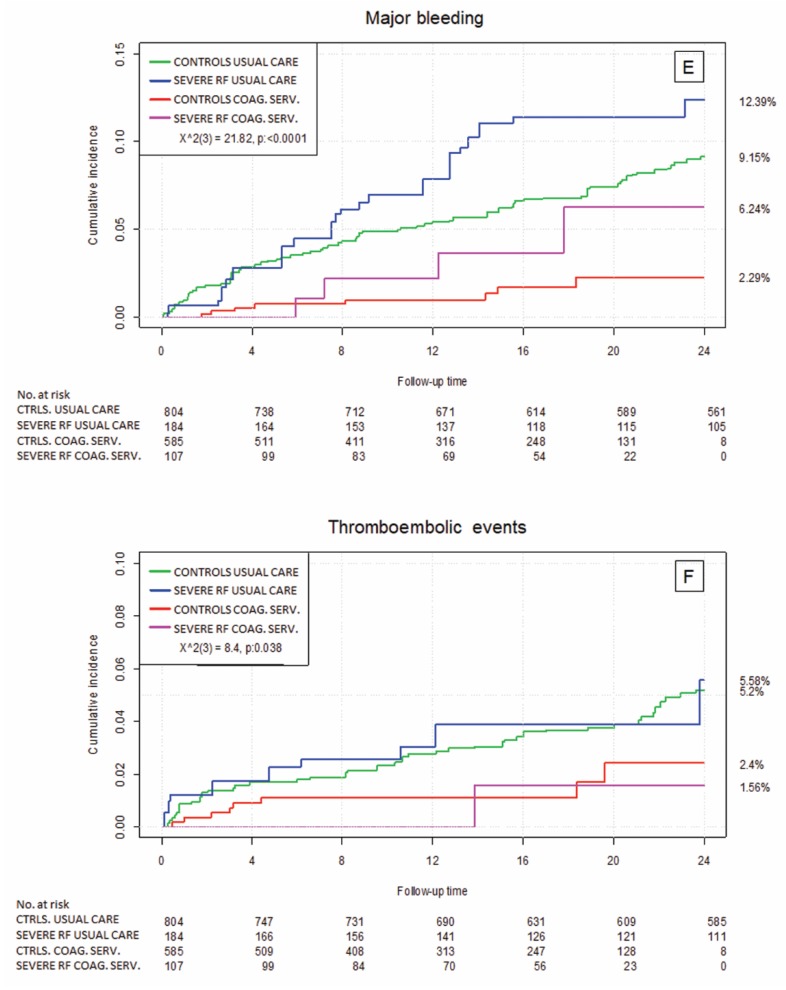
The propensity score adjusted (weighted by inverse probability weights) cumulative incidence plots of the safety endpoints and outcome measures comparing severe renal failure patients and controls in standard care and a coagulation service. (**A**): long-term outcome, (**B**): death, (**C**): hospitalizations, (**D**): all clinically relevant bleeding, (**E**): major bleeding, (**F**): thromboembolic events; Ctrls. Usual care: patients without severe renal failure (RF) managed in usual care; Severe renal failure usual care: patients with severe renal failure managed in usual care; Ctrls. coag. serv.: patients without severe renal failure managed in a coagulation service; severe RF coag. serv.: patients with severe renal failure managed in a coagulation service.

To account for potential differences in the groups, we used propensity score-weighted cumulative incidence plots to compare all four groups (Figure 3).

The composite long-term outcome (Figure 3A) was better in the coagulation cohort in patients with and without severe renal failure compared to the usual care cohort. The cumulative incidence of death was comparable in usual care patients without severe renal failure and coagulation-service- (CS) managed patients with severe renal failure (Figure 3B). Hospitalizations, major bleeding, all clinically relevant bleeding, and thromboembolic events (Figure 3C–F) were lower in coagulation-service-managed patients, irrespective of the absence or presence of severe renal failure. Overall, patients fared significantly better if managed in a specialized coagulation service. The average follow-up was shorter in the coagulation service. We accounted for this by repeating the statistical analysis with censoring events after 24 months of follow-up time. This did not affect the results.

## 4. Discussion

This is the first and largest study to show that patients who require oral anticoagulation therapy benefit substantially from being cared for in a specialized coagulation service, regardless of their renal failure status.

In the present analysis, patients with severe renal failure had a significantly worse outcome compared to patients without severe renal failure during a long-term follow-up period of 3 years. Even after correction for clinically relevant confounding variables, including antiplatelet use, severe renal failure patients had a significantly worse long-term outcome, which, in our study, was a composite endpoint of clinically-relevant non-major and major bleeding, myocardial infarction, systemic and pulmonary embolism, stroke, and death. Furthermore, as single endpoints, severe renal failure patients experienced significantly more deaths and hospitalizations compared to patients without severe renal failure. Severe renal failure proved to be an independent negative prognostic factor. In general, as has been shown by Prochaska et al., managing patients requiring oral anticoagulation in a specialized coagulation service improved their prognosis [20]. We showed that even patients with severe renal failure benefitted substantially from being managed in a specialized coagulation service. Patients with renal failure managed in a specialized coagulation service even outperformed patients without severe renal failure in usual care in their long-term outcome.

It is well-known that patients with chronic kidney disease have a higher incidence of atrial fibrillation and, in general, a lower life expectancy [9]. The benefit of oral anticoagulation has been clearly established for atrial fibrillation patients with an increased risk of stroke and normal renal function [1]. However, considering the high number of patients with chronic kidney disease, the collected evidence favoring oral anticoagulation in this patient population is sparse. There is a general perception that renal failure patients have a worse outcome. However, to our knowledge, large randomized studies researching the quality and outcome of oral anticoagulation in severe renal failure patients are not available because renal impairment is usually an exclusion criterion in these trials. 

Patients with severe renal failure are known in general to have a lower time in therapeutic range [21,22], which is in good agreement with our data. In patients with normal renal function, a higher TTR was associated with a better outcome but not with a significant difference in clinically relevant bleeding events [23]. In a large Danish registry, patients with renal failure have a lower TTR and more thromboembolic events compared to patients with normal renal function [22]. Counterintuitively, TTR did not significantly alter outcomes in our study. Interestingly, in our study, patients cared for in the specialized coagulation service had significantly fewer INR fluctuations, which might explain some of the differences. However, further research is needed to prove this hypothesis.

The risk of death was much greater in severe renal failure patients, which was attenuated by signing these patients up with a specialized coagulation service. In our study, managing severe renal failure patients in a coagulation service reduced the complication rates of these patients down to the complication rates of patients without severe renal failure in usual medical care.

The results of our study further emphasize the fact that patients with severe renal failure are a very particular subgroup of patients, characterized by a distinct clinical risk profile. Consequently, the generated study data, excluding this subgroup, cannot be extrapolated to patients with severe renal failure. Even well-established therapies, such as statin therapy in coronary artery disease, do not appear to be effective in chronic hemodialysis patients due to the altered lipid profile of such patients [13]. An interdisciplinary effort is necessary to research the impact of renal failure on established treatments for cardiovascular and cerebrovascular disease. Different stages of renal failure might require different anticoagulation treatment strategies. At present, there is no clear-cut parameter to facilitate the decision to initiate oral anticoagulation in severe renal failure patients. Traditional scores like the CHADS_2_- or CHA_2_DS_2_-VAsc-score exclude renal failure, and efforts to include renal failure in the calculation have not succeeded [24]. Furthermore, there is accumulating evidence that NOACs exhibit a superior safety and efficacy profile over VKAs in patients with chronic kidney disease [25].

This study has several limitations that merit consideration. First, the study has an all-comer observational design and does not include an intervention. Conversely, this might also represent a strength of the study, as it better approximates real-life situations. There were no scheduled, on-site patient visits; instead, patients were followed up extensively on an annual basis with patient telephone interviews, and checking with family doctors and the residents’ registration office if patients deceased. Second, the thrombEVAL study was not specifically designed to research renal failure patients. Third, assigning the patients of our study to glomerular filtration rate-based strata is not possible since laboratory data on renal function were not recorded. However, currently, a generally accepted, universal definition of renal failure for clinical trials does not seem to exist, as what is considered renal failure varies greatly in most clinical trials or registries. We advocate inaugurating such a definition or the classification of renal failure for clinical trials to allow for a better comparison of renal failure populations among clinical trials. Although our study did not exclude any ethnicities, the study population is mainly Caucasian, and the results may not extrapolate to populations of other ethnic backgrounds. Finally, the generalizability of the findings to other pharmacological agents used for oral anticoagulation therapy (e.g., NOACs) should be done with caution.

## 5. Conclusions

Patients with severe renal failure have a significantly poorer long-term outcome, an increased rate of hospitalization and death, and showed a tendency to have more bleeding events. Managing patients in a specialized coagulation service resulted in substantial risk reductions across all relevant domains, to the extent that patients with severe renal failure in the coagulation service had better outcomes than patients without severe renal failure receiving usual medical care. Accordingly, patients with severe renal failure should be managed in a specialized coagulation service.

## Figures and Tables

**Table 1:** The baseline characteristics of the study sample comparing patients with severe renal failure and controls in usual medical care.

	Controls (*n* = 1183)		Severe Renal Failure (*n* = 333)		*p*
Age	73.0	(64.0/78.9)	75.0	(68.0/81.0)	<0.001
Male sex	63.2%	(748/1183)	65.8%	(219/333)	0.40
CHA_2_DS_2_-VASc	3.95	(1.78 SD)	4.72	(1.59 SD)	<0.001
HAS-BLED	2.57	(1.18 SD)	3.93	(1.04 SD)	<0.001
Charlson index	5.54	(2.23 SD)	6.97	(2.26 SD)	<0.001
Care level present	5.5%	(64/1171)	9.8%	(32/328)	0.007
**Traditional CV risk factors**					
Diabetes	26.8%	(317/1183)	45.6%	(152/333)	<0.001
Dyslipidemia	53.0%	(626/1181)	62.2%	(207/333)	0.003
FH of MI/Stroke	40.5%	(479/1182)	41.4%	(138/333)	0.80
Hypertension, any grade	76.7%	(907/1182)	86.2%	(287/333)	<0.001
Obesity	29.7%	(459/1183)	32.2%	(107/332)	0.28
Smoker, current	8.3%	(98/1183)	5.7%	(19/333)	0.13
**Comorbidities**					
Atrial Fibrillation	71.5%	(842/1177)	80.9%	(266/329)	<0.001
Coronary Artery Disease	40.6%	(459/1131)	49.4%	(157/318)	0.006
Myocardial Infarction	20.3%	(238/1175)	27.4%	(90/329)	0.007
Heart Failure, any grade	37.3%	(433/1161)	62.4%	(204/327)	<0.001
History of Bleeding	30.3%	(342/1129)	38.3%	(118/308)	0.009
History of DVT ^a^	17.2%	(202/1175)	17.5%	(58/332)	0.93
History of PE ^a^	11.2%	(132/1181)	9.6%	(32/332)	0.48
History of stroke/TIA	16.1%	(190/1179)	20.2%	(67/331)	0.082
Peripheral Arterial Disease	22.3%	(257/1151)	25.8%	(84/326)	0.21
Chronic Lung Disease	18.6%	(218/1170)	34.1%	(113/331)	<0.001
Sleep Apnea	9.3%	(105/1126)	10.1%	(32/317)	0.67
Autoimmune Disease	8.2%	(95/1165)	11.1%	(37/332)	0.10
Liver Disease	4.2%	(49/1177)	10.6%	(35/329)	<0.001
Mental Illness	10.4%	(122/1177)	12.0%	(40/332)	0.37
Neoplasm	15.4%	(181/1173)	20.6%	(68/330)	0.029

CV: cardiovascular, FH: family history; ^a^: not exclusive; DVT: deep vein thrombosis; PE: pulmonary embolism; *p* < 0.05: statistically significant difference; SD: standard deviation, numbers in round brackets: the number of cases/the total number of cases with complete information.

**Table 2:** Indication for oral anticoagulation comparing patients with severe renal failure and controls managed in usual medical care.

	Controls (*n* = 1183)		Severe Renal Failure (*n* = 364)		*p*
Atrial Fibrillation	64.6%	(764/1183)	72.1%	(240/333)	0.011
Venous Thromboembolism	13.6%	(161/1183)	7.2%	(24/333)	0.001
Peripheral Bypass	10.7%	(126/1183)	6.0%	(20/333)	0.011
Mechanical Heart Valve	9.1%	(108/1183)	10.2%	(34/333)	0.53
Embolism	7.9%	(93/1183)	4.5%	(15/333)	0.04
Thrombosis	5.7%	(68/1182)	2.7%	(9/333)	0.024
Other	4.5%	(53/1183)	6.0%	(20/333)	0.25

*p* < 0.05: statistically significant difference; numbers in round brackets: the number of cases/the total number of cases with complete information.

## Supplementary Materials

The following are available online at https://www.mdpi.com/2077-0383/9/3/645/s1, Table S1: Baseline characteristics of the study sample comparing severe renal failure patients and controls managed in a specialized coagulation service. Figure S1: Flow diagram showing patient selection. Figure S2. Unadjusted safety endpoints and outcome measures comparing severe renal failure patients in a coagulation service with those receiving usual medical care. Figure S3: Adjusted safety endpoints and outcome measures comparing usual medical care and coagulation service-managed patients, Cox regression.
