# Supplementary material for: Specialized Management of Oral Anticoagulation Therapy Improves Outcome in Patients with Chronic Renal Insufficiency
Source: J Clin Med. 2020 Feb 28;9(3):645. doi: 10.3390/jcm9030645 (PMC7141283; doi:10.3390/jcm9030645)
Supplement: Supplementary file 1 [file jcm-09-00645-s001.zip › jcm-718334-Table S1.docx]

**Table S1.** Baseline characteristics of the study sample comparing severe renal failure patients and controls managed in a specialized coagulation service.

|  | **Controls (*n* = 638)** | | **Severe renal failure (*n* = 118)** | | ***p*** |
| --- | --- | --- | --- | --- | --- |
| Age | 72.0 | (62.9/79.0) | 77.5 | (71.0/83.1) | **<0.0001** |
| Male sex | 51.7% | (330/638) | 53.5% | (62/118) | 0.92 |
| CHA_2_DS_2_-VASc | 3.82 | (1.73 SD) | 4.82 | (1.75 SD) | **<0.0001** |
| HAS-BLED | 2.40 | (1.20 SD) | 3.79 | (1.18 SD) | **<0.0001** |
| Charlson Index | 5.11 | (2.38 SD) | 6.48 | (2.21 SD) | **<0.0001** |
| Care Level Present | 7.7% | (49/637) | 16.9% | (20/118) | **0.0028** |
| **Traditional CV risk factors** | | | | | |
| Diabetes | 25.6% | (162/633) | 29.1% | (34/117) | 0.43 |
| Dyslipidemia | 40.8% | (260/638) | 46.6% | (55/118) | 0.26 |
| FH of MI/stroke | 30.7% | (196/638) | 28.0% | (33/118) | 0.59 |
| Hypertension, any grade | 73.8% | (471/638) | 84.7% | (100/118) | **0.010** |
| Obesity | 32.0% | (204/638) | 29.2% | (35/118) | 0.67 |
| Smoker, current | 6.0% | (38/638) | 2.5% | (3/118) | 0.18 |
| **Comorbidites** | | | | | |
| Atrial Fibrillation | 61.9% | (394/637) | 72.9% | (86/118) | **0.022** |
| Coronary Artery Disease | 25.2% | (160/635) | 41.9% | (49/117) | **0.00045** |
| Myocardial Infarction | 10.7% | (68/633) | 19.7% | (23/117) | **0.013** |
| Heart Failure, any grade | 28.9% | (183/634) | 40.2% | (47/117) | **0.017** |
| History of bleeding | 16.0% | (102/637) | 15.3% | (18/118) | 0.89 |
| History of DVT^a^ | 27.6% | (175/634) | 24.8% | (29/117) | 0.57 |
| History of PE^a^ | 16.2% | (103/637) | 21.2% | (25/118) | 0.18 |
| History of stroke/TIA | 16.1% | (103/638) | 23.7% | (28/118) | 0.063 |
| Peripheral Arterial Disease | 10.1% | (64/636) | 14.4% | (17/118) | 0.19 |
| Chronic Lung Disease | 13.7% | (87/636) | 25.4% | (30/118) | **0.0022** |
| Sleep Apnea | 6.7% | (42/627) | 13.0% | (15/115) | **0.034** |
| Autoimmune Disease | 7.5% | (48/636) | 6.8% | (8/118) | 1.0 |
| Liver Disease | 3.6% | (23/637) | 2.5% | (3/118) | 0.78 |
| Mental Illness | 9.7% | (62/636) | 11.0% | (13/118) | 0.62 |
| Neoplasm | 16.9% | (106/627) | 23.9% | (28/117) | 0.087 |

CV; cardiovascular; FH: family history; ^a^: not exclusive; DVT: deep vein thrombosis; PE: pulmonary embolism; *p* < 0.05: statistically significant difference (bold); SD: standard deviation, numbers in brackets: number of cases/number of cases with completely reported information.
