# Supplementary figures and images for: Specialized Management of Oral Anticoagulation Therapy Improves Outcome in Patients with Chronic Renal Insufficiency
Source: J Clin Med. 2020 Feb 28;9(3):645. doi: 10.3390/jcm9030645 (PMC7141283; doi:10.3390/jcm9030645)

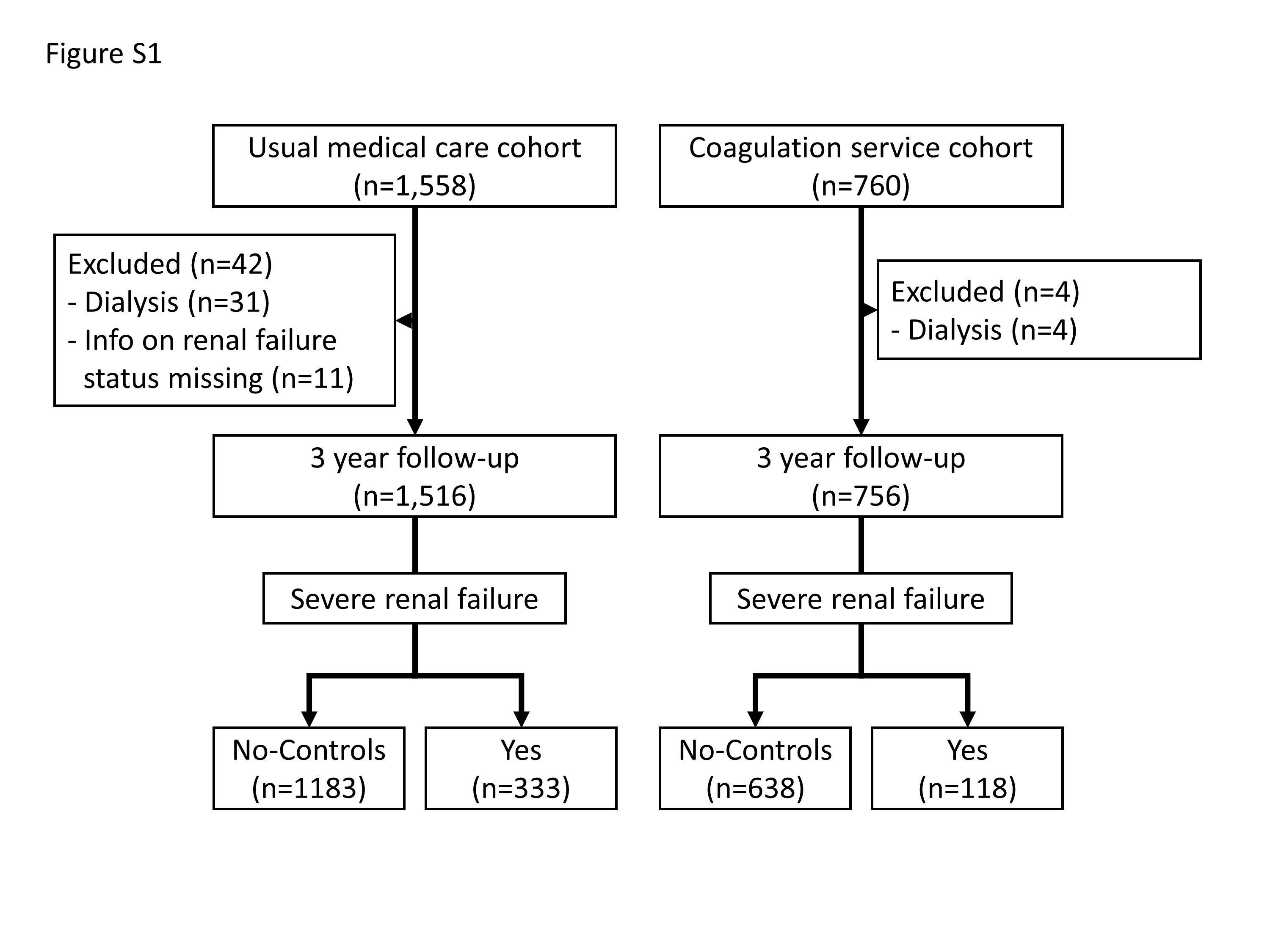

Supplement: Supplementary file 1 [file jcm-09-00645-s001.zip › jcm-718334 Figure S1.tif]

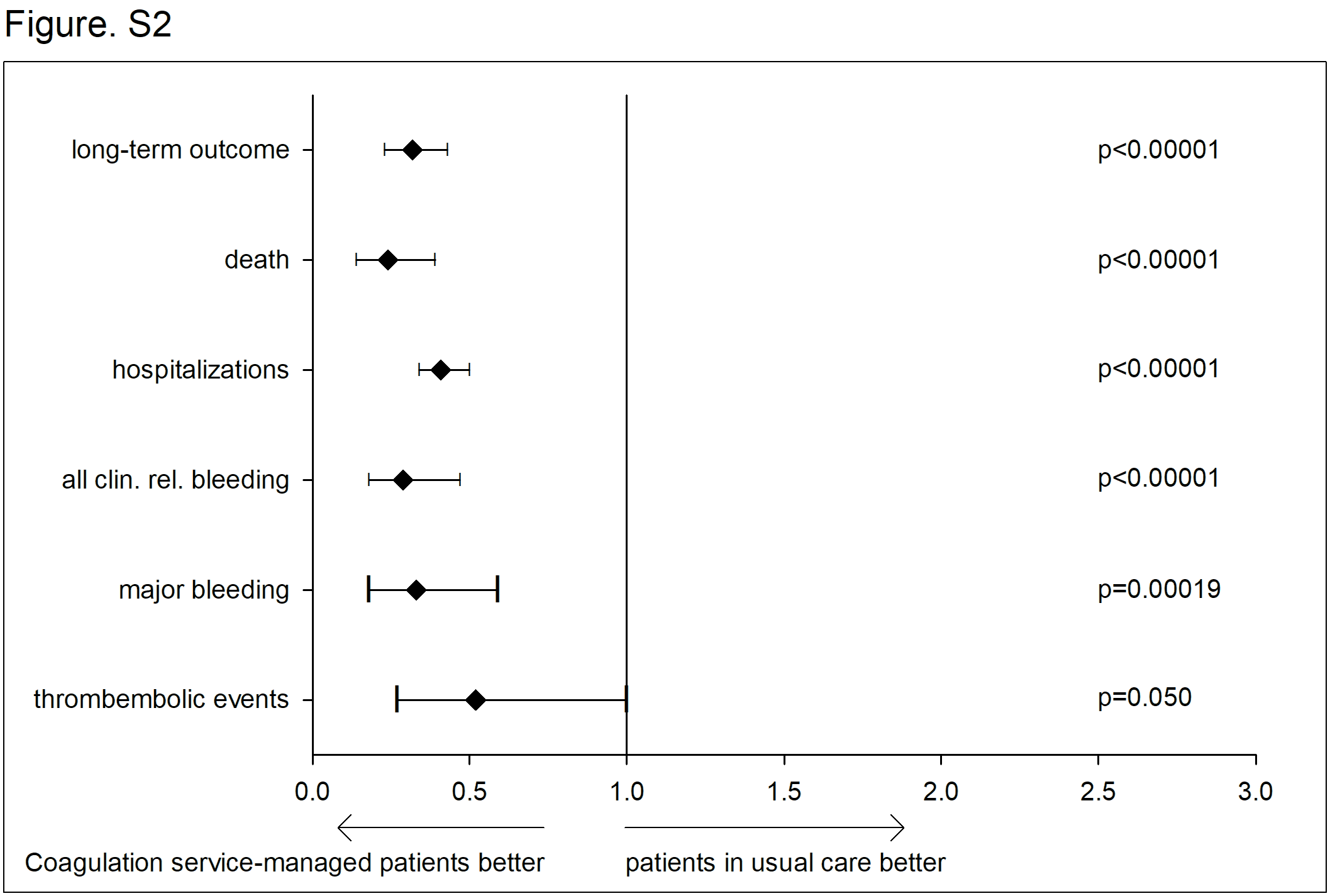

Supplement: Supplementary file 1 [file jcm-09-00645-s001.zip › jcm-718334 Figure S2.tif]

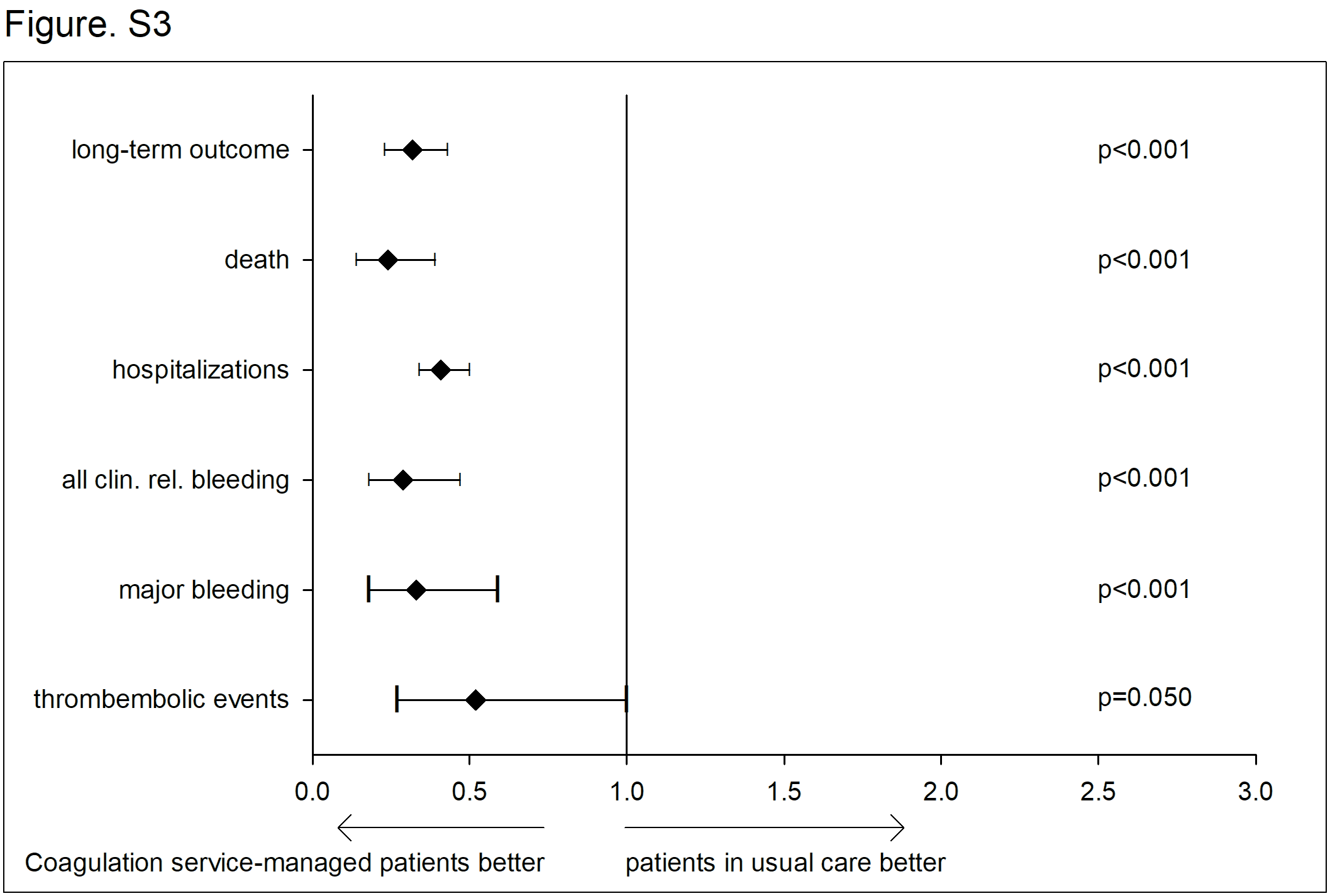

Supplement: Supplementary file 1 [file jcm-09-00645-s001.zip › jcm-718334 Figure S3.tif]
